# Suprapubic catheter change: Evaluating YouTube videos as a resource for teaching junior doctors

**DOI:** 10.1002/bco2.299

**Published:** 2023-10-20

**Authors:** Thomas Milton, Peter Stapleton, Darcy Noll, Shrirajh Satheakeerthy, Joseph Hewitt, Ashani Couchman

**Affiliations:** ^1^ University of Adelaide Adelaide South Australia Australia; ^2^ Department of Surgery Royal Adelaide Hospital Adelaide South Australia Australia; ^3^ Young Urology Researchers Organisation Melbourne Victoria Australia

**Keywords:** DISCERN, JAMA, SPC change, suprapubic catheter change, YouTube

## Abstract

**Objectives:**

The objectives of this study are to assess the current level of experience and teaching practices for SPC change at our institution and, second, to assess the quality of YouTube videos as an educational tool for teaching SPC change.

**Methods:**

A survey was conducted of 40 JMOs at our institution regarding SPC change. The first 20 YouTube videos on SPC change were included for analysis. A JAMA and DISCERN score was calculated for each video. Using linear regression, the association between collected variables and the assigned JAMA and DISCERN scores were determined.

**Results:**

The survey showed that 18 (45%) of JMOs had done an SPC change. None had received formal teaching. The consensus was that the quality of the YouTube videos was poor. There was a statistically significant positive correlation between the score assigned to videos by each scoring system (Pearson's *r* 0.81, *p <* 0.001). There was no statistically significant association between video quality as measured by either of the scoring systems and number of views. No association between any video characteristic and JAMA and DISCERN score was found.

**Conclusion:**

An SPC change is often a requirement of JMOs; however, this skill is not formally taught. The quality of YouTube videos describing an SPC change is poor.

## 1 INTRODUCTION

A suprapubic catheter (SPC) change is a common procedure. Given how common and how simple it is, an SPC change should be a skill that all junior medical officers (JMO) are able to perform. Lack of experience and confidence is likely responsible for many JMOs not being able to perform an SPC change.

YouTube is the most commonly used video website in the world.^1^ A survey conducted in 2016 of medical students who were pursuing a career in surgery found that YouTube was the most frequently used video educational resource for surgical preparation, with 86% reporting to have used YouTube.^2^ Videos on YouTube do not undergo a peer review process. This may prove to be problematic as junior doctors using these videos as an educational tool may not have the ability to critically appraise the video compared to an experienced doctor. Previous studies have found that the quality of YouTube videos in healthcare is poor and that there are not any clear variables that help predict its quality.^3^, ^4^, ^5^, ^6^, ^7^, ^8^

The objectives of this study were first to assess the current level of experience and teaching practices for SPC change at our institution. Second, the quality of YouTube videos was assessed as a potential educational tool for JMOs.

## 2 MATERIALS AND METHODS

A survey was distributed via email to JMOs at our health network. Data were collected regarding doctors' previous experience in changing an SPC and on what teaching they received to enable them to perform this procedure (see Supporting Information S1).

YouTube was searched for ‘how to perform a suprapubic catheter change’ on the 25th of February 2023, and the first 20 video results were included in the study for analysis. Videos were excluded if they were not in English or did not demonstrate how to perform an SPC change. For each of the videos, the following was recorded: number of views, date posted, date accessed, how many days since the video was posted, the video length, the number of likes, the number of likes per view, the number of comments, and the author category. The author category was divided into healthcare worker and patient testimony.

Two authors independently calculated a JAMA^9^ and DISCERN^10^ score for each of the videos. The JAMA score is calculated by giving a score out of four to the category's authorship, attribution, currency, and disclosure. The DISCERN score is obtained through a 15‐part questionnaire with each question out of 5 points giving a mean score at the end, which has assessed its reliability and quality.

Intraclass correlation (ICC) was used to assess interobserver reliability for both the scoring systems, with values of >0.6 considered as good correlation. Using linear regression, the association between collected variables and the assigned JAMA and DISCERN score was determined. After controlling for the age of the video, linear regression was used to determine the relationship between number of views and comments and the assigned score. All statistical analysis was completed using Jamovi.

The study was reviewed by the Central Adelaide Local Health Network Human Research Ethics Committee and approved as a quality improvement study (approval number: 17859). Requirement to obtain formal written consent was waived, and participation in the voluntary online survey was taken as consent by study participants.

## 3 RESULTS

**FIGURE 1:**
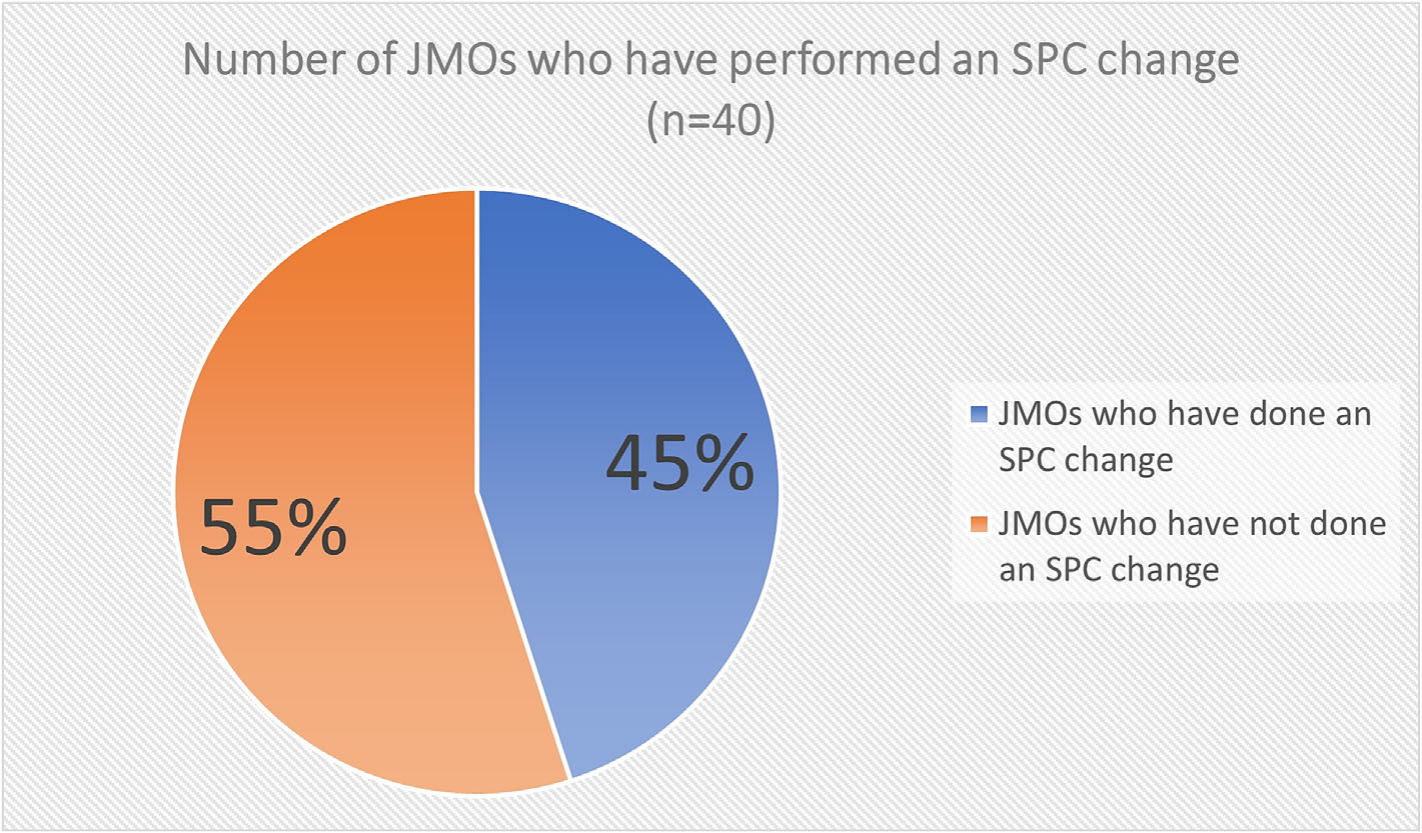
Number of JMOs who have performed an SPC change (*n* = 40).

**FIGURE 2:**
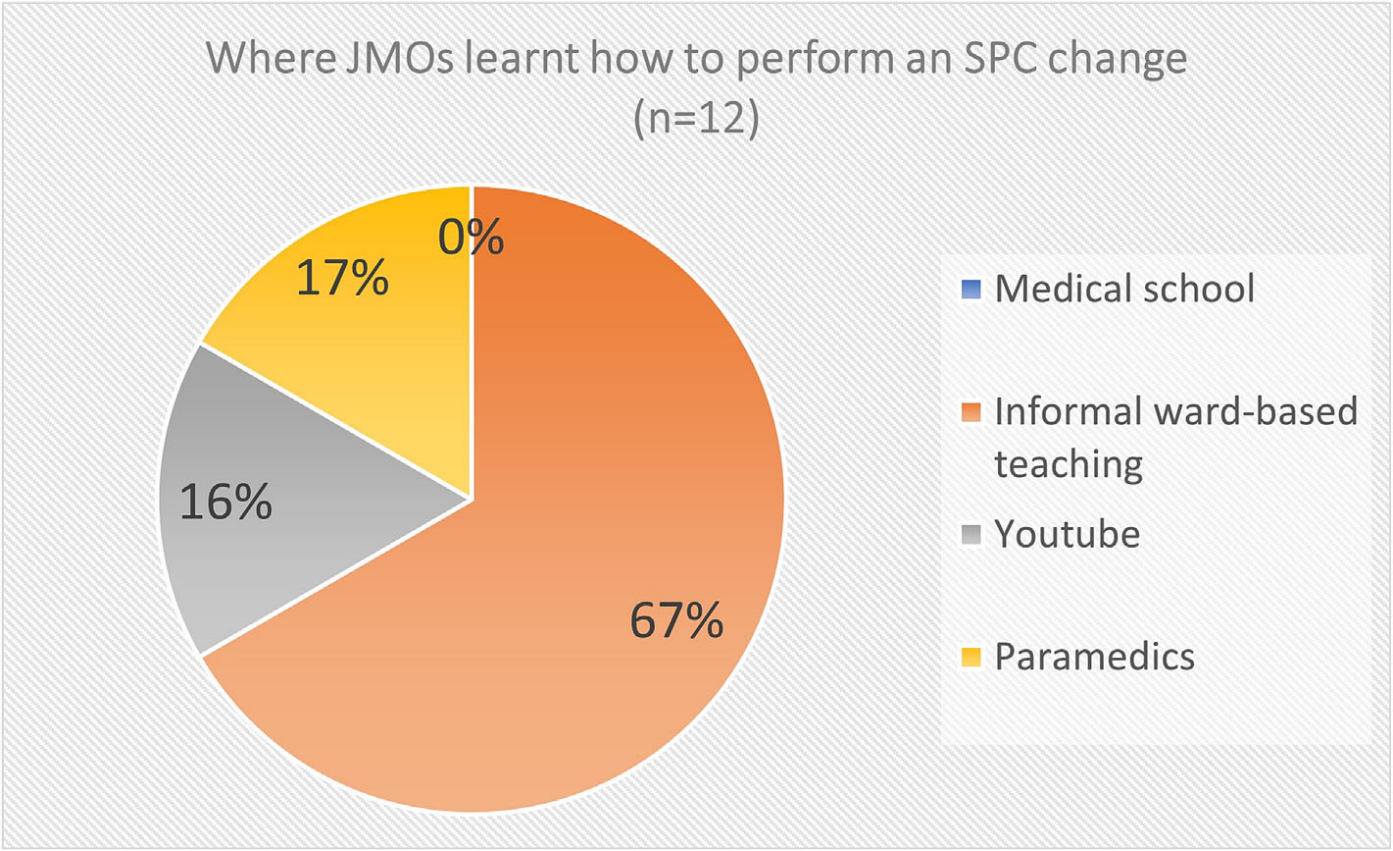
Where JMOs learnt how to perform an SPC change (*n* = 12).

**FIGURE 3:**
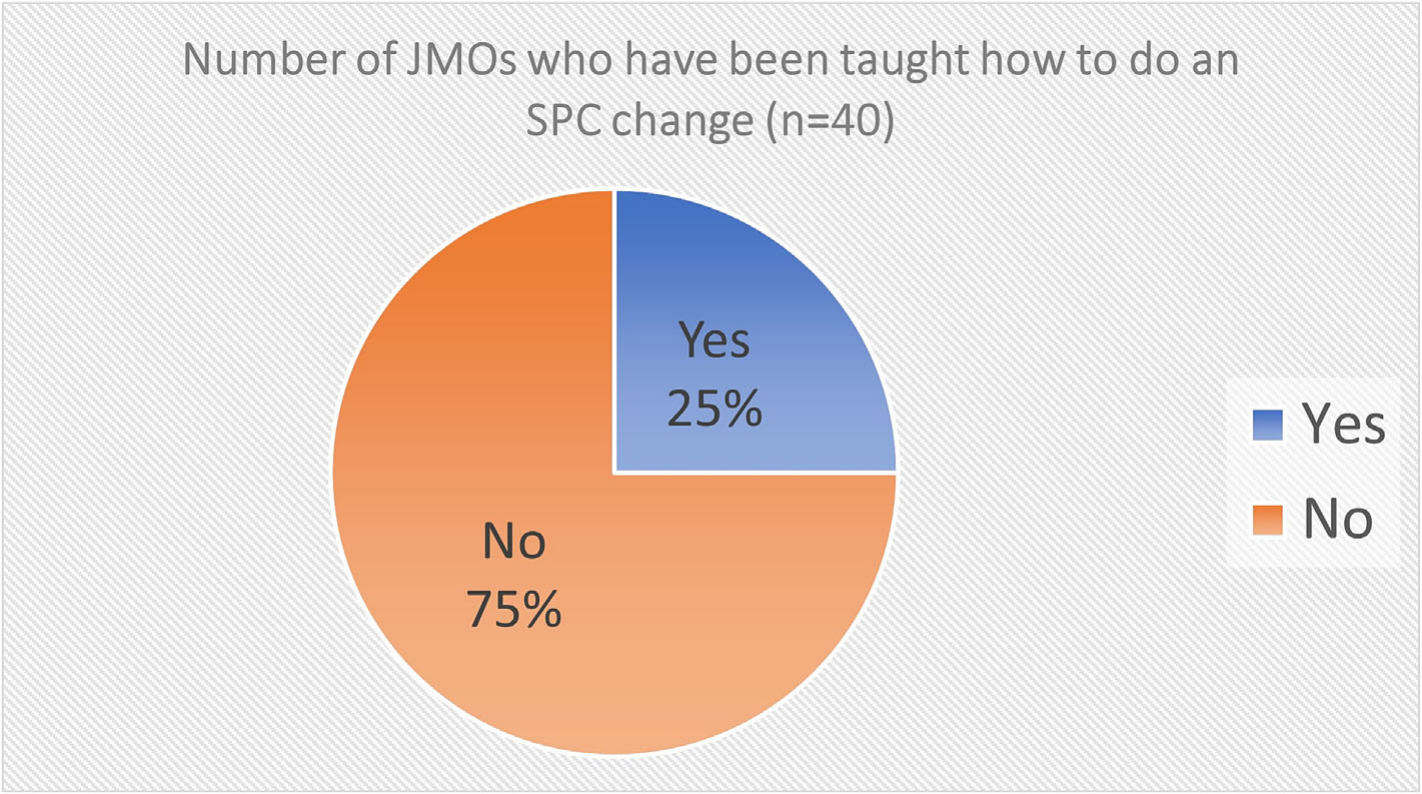
Number of JMOs who have been taught how to do an SPC change (*n* = 40).

Forty responses were received to the survey of JMOs. Eighteen (45%) of the JMOs had performed an SPC change (Figure 1). Of this group, seven (39%) were taught informally on the ward, two (11%) learned from YouTube, one (6%) learned from previous employment as a paramedic, and eight (44%) had never been taught (Figure 2). Overall, there were 10 (25%) JMOs who reported they had received some form of teaching to do an SPC change (Figure 3). All of the 22 (56%) who had not done an SPC change reported they had never been taught. No one was taught how to do an SPC change in medical school or through a formal tutorial.

**FIGURE 4:**
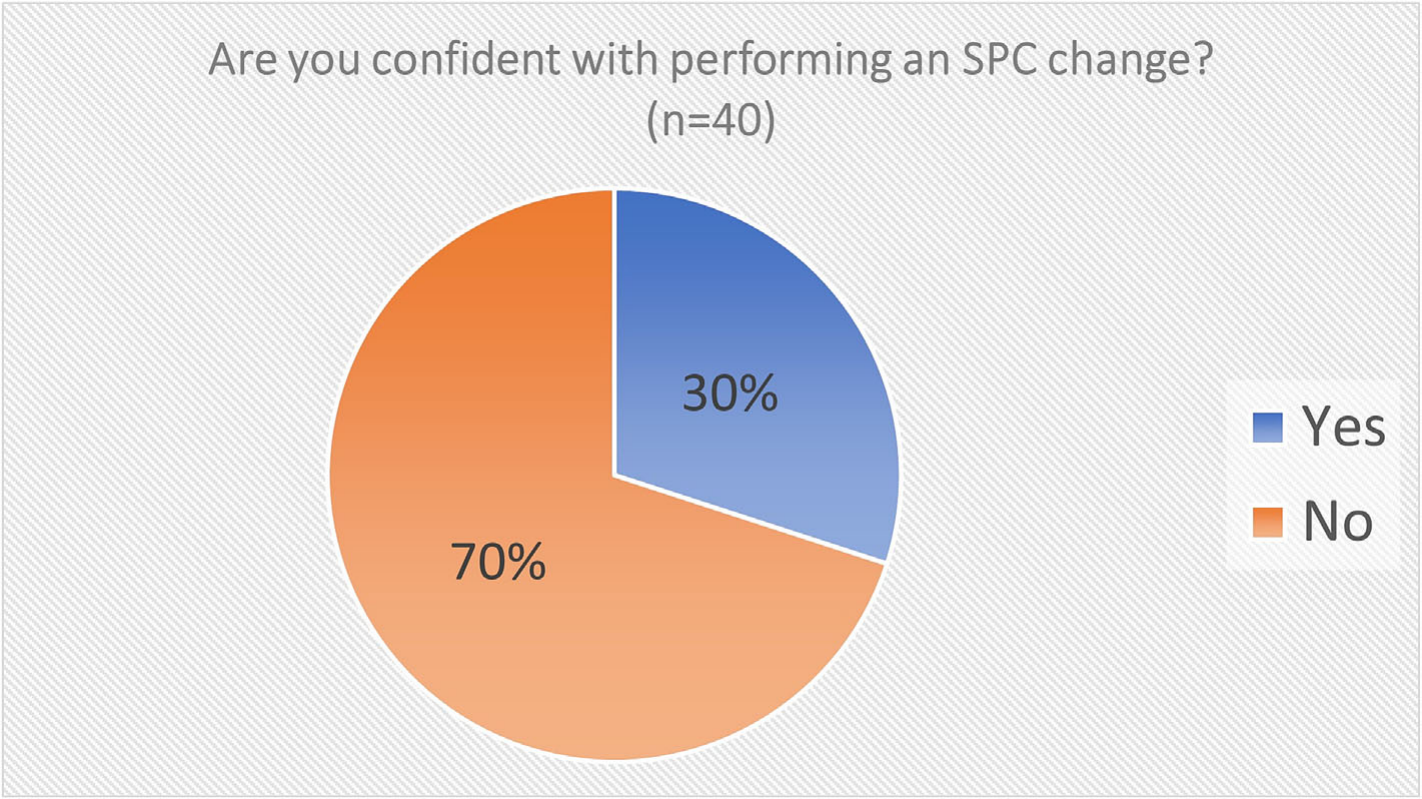
Are you confident with performing an SPC change (*n* = 40)?

There were 12 (30%) JMOs who reported they were confident performing an SPC change compared with 28 (70%) who were not confident (Figure 4). All of the 12 who reported they were confident also said that they had done an SPC change previously. However, six people who said they were not confident doing an SPC change had reported they had done one previously. Everyone who reported they were not confident had not done an SPC change previously.

Of the 20 videos evaluated from YouTube, the median number of views was 16 029 with an interquartile range (IQR) 2489–73 761. The median number of likes was 22 with an IQR 6–254. The median number of days online was 1552 with an IQR 1052–2567. The median consensus JAMA score was 1.5 with an IQR 1–2.5, and the median consensus DISCERN score was 2 with an IQR 1–2.5.

The consensus was that the quality of the YouTube videos was poor. ICC coefficients were 0.68 for JAMA and 0.60 for DISCERN, indicating good correlation between observers for each scoring system.^11^ There was a statistically significant positive correlation between the score assigned to videos by each scoring system (Pearson's *r* 0.81, *p* < 0.001).

**FIGURE 5:**
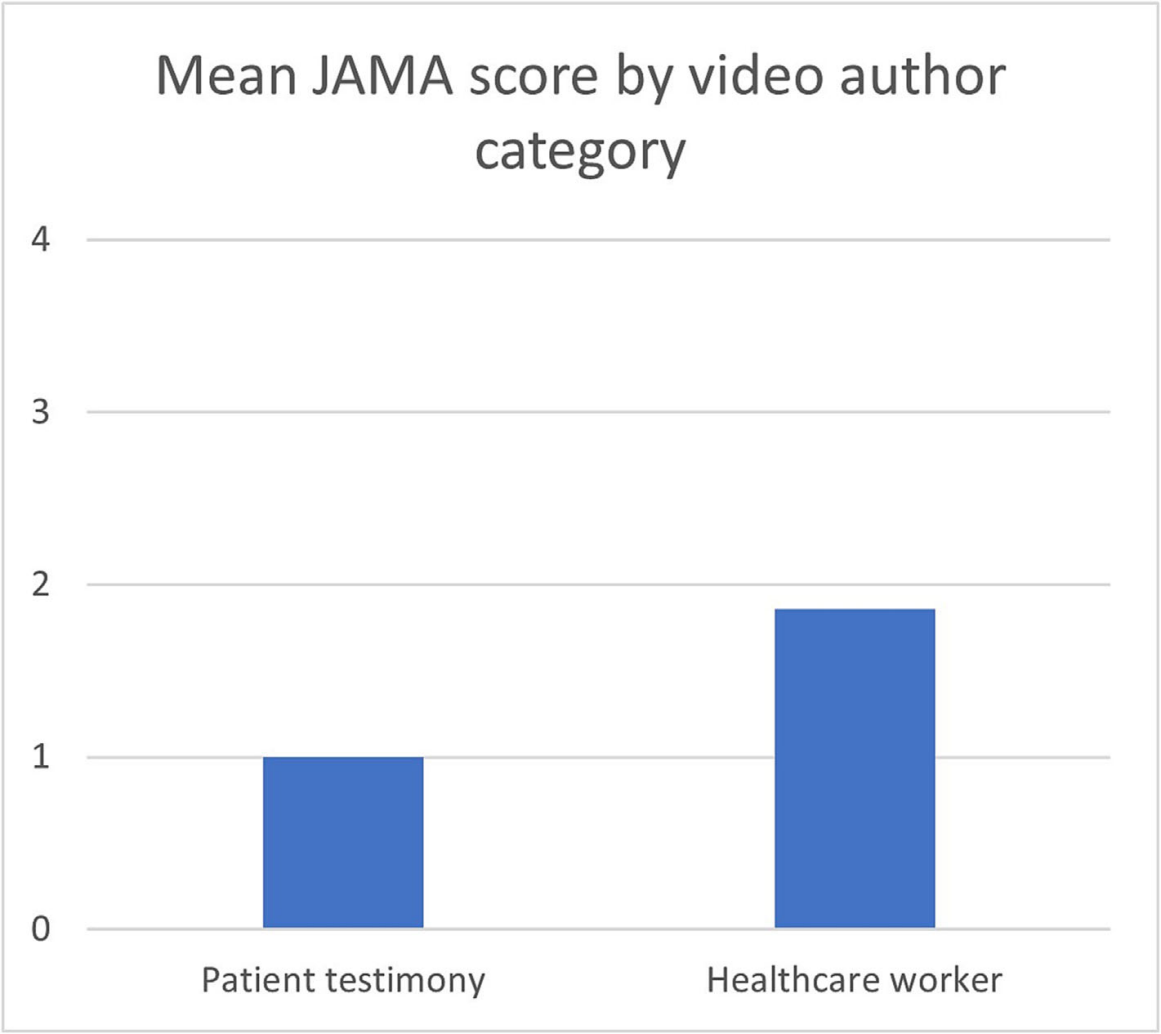
Mean JAMA score by video author category.

**FIGURE 6:**
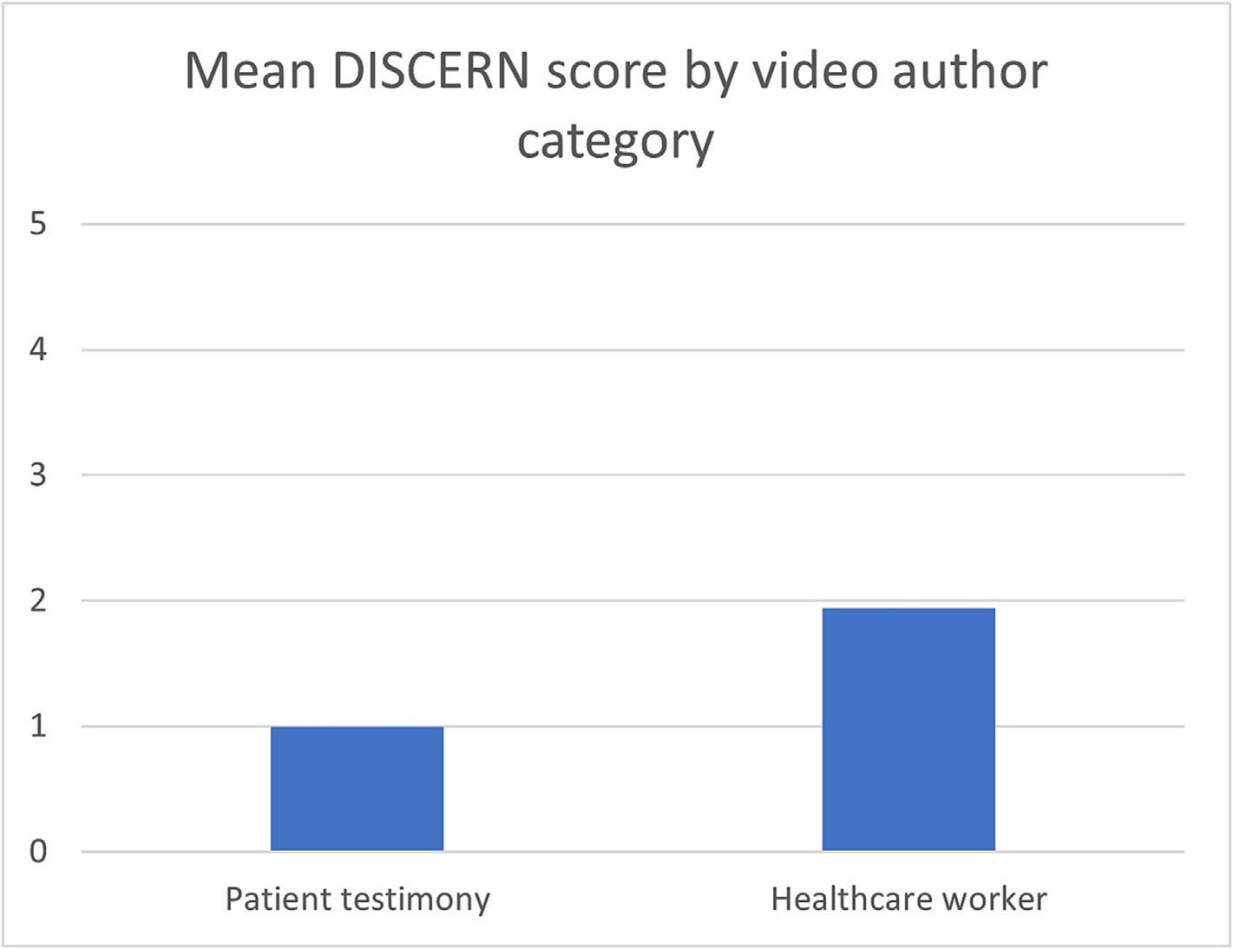
Mean DISCERN score by video author category.

There was no statistically significant association between video quality as measured by either of the scoring systems and number of views. This result was the same when the number of days online was included as a covariate. When considering the author of the video, the videos produced by a healthcare worker had an average JAMA score of 1.86/5 and DISCERN score of 1.98/5, compared to patient testimony which scored a 1/4 in JAMA and 1/5 DISCERN (Figures 5 and 6). This result demonstrates that videos created by healthcare workers scored higher than those created by patient testimony, but both were still considered to be of poor quality.

## 4 DISCUSSION

The survey demonstrated that SPC change is a skill commonly performed by JMOs in our health system but that teaching the skill is lacking or non‐existent. Without formal teaching being available, and the requirement for a significant proportion of JMOs to do an SPC change, this demonstrates the value in having an online video demonstration as an easy way to teach SPC change. The question remains to be answered as to whether or not YouTube can be an appropriate way for this to be delivered. It is somewhat concerning that the results of this study have found the quality of YouTube videos for SPC change is generally poor.

This result was somewhat expected, given similar studies in healthcare that have previously evaluated the quality of YouTube videos mostly found them to be of poor quality.^4^, ^5^, ^6^, ^7^, ^8^ A systematic review in 2022 by Gorgy et al.^12^ aimed to review the studies that have done a quality assessment of YouTube videos in healthcare and found that 96.6% reported that the quality was unsatisfactory.

There were seven studies that evaluated the quality of YouTube videos specific to Urology. Of these studies, five of them reported the quality of YouTube videos was low, consistent with this study and from the systematic review by Gorgy et al.^4^, ^13^, ^14^, ^15^, ^16^ Of these five studies, three generated their own criteria for evaluating YouTube videos, one of them used their own criteria and the LaP‐VEGaS criteria and one created criterion based off of the EAU guidelines and ‘established clinical practice’ for clean intermittent self‐catheterisation (CISC). None of these used the JAMA or DISCERN score. For all of these studies, there was no consistent scoring system used to evaluate the quality of YouTube videos.

A study by Culha et al. that evaluated the quality of YouTube videos for CISC actually found that the majority of YouTube videos on CISC training videos contained useful information. They judged the content subjectively by having two independent experts in the field review the video and then calculated a DISCERN score and global quality score (GQS). It also found that the majority of the videos were produced by for‐profit companies, which was not the case for this study. It found that none of the videos produced by non‐healthcare worker individuals were useful.^17^

The final study evaluating YouTube videos in Urology, which looked at male urethral catheterisation, had slightly different methods. In this video, criterion was developed to evaluate the YouTube videos; however, only the top‐rated videos were then showed to junior doctors as an educational tool. They similarly found that the quality of YouTube videos is widely variable, but when choosing just the highest rated videos, all of the junior doctors reported they were a useful adjunct to their education.^18^

The systematic review by Gorgy et al.^12^ found that there is no standardised approach to evaluate the quality of YouTube videos. Approximately half of the studies reviewed used one of the standardised scoring systems, such as DISCERN, whereas half had an arbitrary subjective score created by the author. Due to the heterogeneity of the scores, it did not allow a quantitative analysis of the studies. Of the 29 studies reviewed in this systematic review, 22 explored the association between variables in the video and the quality. Although some individual studies found an association between specific variables and quality, there was no one variable that consistently demonstrated an association with quality in the majority of the studies. Ten of the studies explored whether there was an association between the author category and subjectively how useful the video was, with six of these studies demonstrating an association; however, there was no association with the quality of the video.^12^

A limitation of this study is there were only 20 videos evaluated, which was indicative of the low number of educational videos on YouTube. However, as these were the top 20 results found, they are also the most relevant to viewers. This video used two objective scores that are well established in evaluating healthcare video content; however, neither are specific to Urology or SPC change, as these scores do not exist. A subjective generated criterion could have been used, but this would increase the risk of bias. The future of YouTube video evaluation should head towards more objective criterion with established scores.

## 5 CONCLUSION

This study demonstrated that YouTube videos describing an SPC change are of poor quality when using the JAMA and DISCERN scores. There was no association between any of the variables of the video and quality. This is consistent with other studies that have looked at the quality of YouTube videos in healthcare.^12^ There is significant heterogeneity in the method to evaluate the quality of YouTube videos in healthcare. Given the popularity of YouTube as an educational tool, a standardised evaluation tool should be generated.

## AUTHOR CONTRIBUTIONS

Thomas Milton wrote the manuscript and reviewed the videos for inclusion. Peter Stapleton and Darcy Noll provided scores for the videos independently. Shrirajh Satheakeerthy conducted the survey. Joseph Hewitt completed the statistics. Ashani Couchman reviewed the manuscript for submission.

## DISCLOSURE

The authors declare we have conflict of interest.

## Supporting information


**Data S1.** Suprapubic Catheter Familiarity.
