# Supplementary material for: Suprapubic catheter change: Evaluating YouTube videos as a resource for teaching junior doctors
Source: BJUI Compass. 2023 Oct 20;5(2):224–9. doi: 10.1002/bco2.299 (PMC10869646; doi:10.1002/bco2.299)
Supplement: Supplementary file 1 — Data S1. Suprapubic Catheter Familiarity. [file BCO2-5-224-s001.docx]

**Supplement 1**

**Suprapubic Catheter Familiarity**

This 1 minute survey aims to gather basic information on procedural familiarity with suprapubic catheter from junior medical officers.

1. Have you ever done an SPC change?
   1. Yes
   2. No
2. Have you been taught how to change an SPC?
   1. Yes
   2. No
3. How did you learn about SPC changes?
   1. Medical school
   2. Informal ward-based teaching
   3. Youtube
   4. Other
4. Are you confident with performing an SPC change?
   1. Yes
   2. No
